# Effect of Beta vulgaris extracts on dysmenorrhea among adolescent girls

**DOI:** 10.6026/97320630018657

**Published:** 2022-07-31

**Authors:** B Mahalakshmi, N Sivasubramanian, Payal Vaghela, Rohit Divya Ganvanthbhai, Gurav Priyanka Rajeshbhai, G Ramalakshmi, D Prakash, Gnanadesigan Ekambaram

**Affiliations:** 1 Nootan College of Nursing, Sankalchand Patel University, Visnagar, Gujarat - 384315, India; 2 College of Nursing, S.G.R.R University, Dehradun, Uttarkhand - 248001, India; 3 Department of Physiology, Nootan medical college and Research Centre, Sankalchand Patel University, Visnagar, Gujarat - 384315, India

**Keywords:** Dysmenorrhea, adolescent girls, Beta vulgaris juice

## Abstract

Dysmenorrhea is painful menstrual periods which are caused by uterine contractions. The ache is commonly felt in the pelvic or lower abdomen around the time menstruation begins. Periods aren't the best time for a woman to feel strong and energized. With all of the blood loss, cramps, and exhaustion, finding enough excitement to get through the day's responsibilities is difficult. Vulgaris Beta Potassium and nitrates, both of which are necessary for blood pressure regulation, are abundant in juice. For energy, only 50ml of beet juice is required. The analysis of data was done by descriptive and inferential statistics. The study identified that 46.66 % had moderate pain, 33.33 % had mild pain and none of them had severe pain for the pre- experimental group. The study result shows that the pre-test mean value for is 5.91 and pre-test SD 0.96. The post-test mean value is 2.86 and post-test SD is 1.04. The mean difference is 3.05. The calculated 't' value is 16.85 is higher than the table value 1.67. The study concluded that Beta vulgaris juice found to be effective non-pharmacological measures to reduce dysmenorrheal among adolescent girls.

## Background:

Adolescence is a phase of transition from infancy to adulthood marked by a surge in physical, endocrine, emotional, and cerebral development, as well as a shift from total dependency to relative independence. Adolescence is a phase of physical and psychological preparation for a girl's safe parenting. Adolescent girls' health has an impact on not only their personal health but also the health of future generations because they are direct reproducers. Girls under the age of 20 make up over a quarter of India's population. Menarche, which is typically associated with difficulties of irregular menstruation, heavy bleeding, and dysmenorrhea, is one of the key physiological changes that occur in adolescent girls. Dysmenorrhea is one of the most prevalent problems that many adolescent females face. Nag (1982) found that 33.5 percent of adolescent girls in India suffer from dysmenorrheal [1]. According to a study conducted in Sweden, more than half of all menstruation women report some discomfort [2]. A prominent obstetrician also stated that approximately 5-10 % of girls in their late teens suffer from severe spasmodic dysmenorrhea, which disrupts their educational and social lives [3]. In India, the true incidence and prevalence of dysmenorrhea have yet to be determined. George and Bhaduri [4] recently concluded that dysmenorrhea (87.87%) is a frequent condition in India. In Sweden, the prevalence was between 2 and 4% [2]. Similar findings in rural married women in Andhra Pradesh were reported by Jayashree and Jayalakshmi [5]. In the United States, dysmenorrhea is thought to be the leading cause of time lost from work and education [6]. Beta vulgaris is frequently cooked, but raw beet juice contains a slew of health advantages and is referred to as a "super food" in current nutritional language. Betaine is the pigment that gives beet juice its rich red and purple hue. Nitrate is abundant in Beta vulgaris juice, which can be converted to nitric oxide (NO) after consumption. Its functions in the human body include lowering blood pressure and increasing oxygen and nutrient delivery to various organs, relaxing smooth muscles, increasing stamina by lowering oxygen during exercise, treating anemia by blood count raise and increased blood circulation and capacity of erythrocytes (red blood cells) to carry oxygen, preventing defects at birth through folate and folic acid, and preventing hypertension [7]. Tom Clifford (2015) stated in his study there has been a recent increase in interest in the biological activity of red beetroot (Beta vulgaris rubra) and its potential utility as a health-promoting and disease-preventing functional food. Beetroot consumption, as a source of nitrate, is a natural way to increase in vivo nitric oxide (NO) availability and has emerged as a viable tool for preventing and controlling illnesses like hypertension and endothelial dysfunction that are linked to poor NO bioavailability. Beetroot is also being investigated as a potential therapeutic treatment for a variety of clinical conditions linked to oxidative stress and inflammation. In vitro and in vivo, its components, particularly the betalain pigments, show substantial antioxidant, anti-inflammatory, and chemo preventive effects. Studies have shown that extracts of beet root prevents menstrual cramp [8]. Therefore, it is of interest to explain the biological activity of beetroot and to assess proof from research that looked at how beetroot supplementation affected inflammation, oxidative stress, cognition, and endothelial function [9].

## Materials and Methods:

The investigation was conducted in an experimental method and Kansa was the location of the study. The present study was approved by the institutional ethics committee and an informed consent was taken from all the subjects after explaining the study procedures and the goal of present study in local language. The experiments followed the amended Helsinki Declaration of 1975 that was revised in 2013. The study's research design is a one-group pre-test post-test pre-experimental design. The hospital and sample was chosen using a non-probability convenience sampling technique. For this trial, 60 adolescent girls were chosen, and they were given 50 mL of Beta vulgaris extract (juice) three times a day for two days to treat dysmenorrhea symptoms. Daily record sheet was provided for maintaining the daily intake. Pre-tests were done on the first day of menstruation to determine the severity, and post-tests were done on the third day evening using the same numerical pain rating scale. SPSS (Statistical Package for Social Sciences) was used (version 17.0) to evaluate descriptive and interstitial statistics like mean, SD, and the chi-square test. A statistically significant P-value of 0.05 was used.

## Results:

Table 1(see PDF) shows demographic characteristics of subjects. Majority of subjects were reported normal weight and no specific change was observed in BMI. Table 2 and Figure 1(see PDF) shows frequency and percentage distribution of pre test and post test scores of experimental group respectively shows that, in the pretest 33 (55%) were in severe pain. Whereas in post test 40 (67 %) were in moderate pain, other changes were mentioned in Table 2 and Figure 1(see PDF). Table 3(see PDF) shows that the average pre test and post test scores on the level of pain among adolescent girls with dysmenorrhea in experimental group is statistically significant (p<0.00001). It shows that extract (juice) of Beta vulgaris consumption has effective in reduction of pain among adolescent girls with of dysmenorrheal in the experimental group. Table 4(see PDF) shows the relationship between frequency of post-test dysmenorrhea and selected demographic characteristics among adolescent girls. It shows significant results among age group (chi square 9.13) and income category (chi square 8.25). No other significant results were reported.

## Discussion:

Beta vulgaris, also known as beets, has been cultivated for over a thousand years. Beetroot and its leaves are both pleasant to eat. The secretion of betalain pigments, such as betanin, gives the beetroot its red or pink colour. Niacin, pantothenic acid, folate, riboflavin, vitamin B6, vitamin B1, thiamine, zinc, magnesium, potassium, vitamin C, phosphorus, and dietary fibre are among the vitamins, minerals, and antioxidants found in Beta vulgaris. Beta vulgaris aids in the conversion of nitrite to nitric oxide, a key vasodilator that has been demonstrated to improve blood flow throughout the body, including the uterus. Beetroots are also high in iron, which is necessary for the health of red blood cells. A glass of Beta vulgaris juice can help battle period-induced lethargy and prevent menstrual cramps. It can help you restore your vigour since beetroot is high in iron, which helps to improve blood count and blood flow. The Effect of Long-Term Beetroot Juice Supplementation on Hematological Parameters - A Pilot Study was conducted by Olga Mizera in 2020 [10]. The aim of this study was to check how a diet and beetroot juice supplementation influences haematological parameters, glutathione peroxidase activity in erythrocytes (GPx), and physical performance in elite fencers over the course of four weeks. During the preparation period, 20 fencers participated in the study. Fencers were tested for fitness VO2 max at baseline (B) and after two phases of dietary recommendations implementation-the first without beetroot juice (D) and the second with 26 g/d of freeze dried beetroot juice supplementation (E) (D&J). They discovered that while beetroot juice does not immediately aid with period symptoms, a glass of it can help you restore some vitality. Beetroots are high in iron, which helps to boost blood count and blood flow, allowing oxygen and nutrients to reach all parts of the body. This allows the woman to cope with the pain and continue with her usual activities [10]. Several studies have found that betalains have strong antioxidant and anti-inflammatory properties in vitro and in a range of in vivo animal models [11-14]. Betalain is the principal bioactive chemical found in red beets, and it belongs to the class of red and yellow pigments. Betanin has ten times the antioxidant activity of tocopherol and three times the antioxidant activity of catechin. The compounds are cationized in a test of linoleate peroxidation by cytochrome c, which increases their affinity for membranes, which is a very useful property for antioxidants [15]. Increased level of antioxidants might also be a reason for reduction of the pain during menstruation. In 2010, a group of researchers from Poland found that consuming betalains (35 - 100 mg capsules, twice daily for 10 days), which are the purple/violet nutrients that give beetroot its resplendent colour, reduced joint pain in individuals presenting with symptoms of osteoarthritis [16]. Another statement from medindia.net, intake of 75 ml of a blend of parsley juice with beet, carrot and cucumber juices can reduce menstrual cramps [17].

Beetroot juice is used as a supplement because of its high inorganic nitrate (NO3−) Once ingested, the NO3− is reduced to nitrite (NO2−), by anaerobic bacteria in the oral cavity by the action of nitrate reductase enzymes and then to nitric oxide (NO) in the stomach. Nitric oxide (NO) is involved in many physiological processes and several lines of evidence have indicated that NO plays a complex and diverse role in the modulation of pain. Nitric oxide is an important neurotransmitter involved in the nociceptive process and, in the dorsal horn of the spinal cord; it contributes to the development of central sensitization [18-22]. It indicates that increased NO might have played an important role in reduction of dysmenorrheal. Further detailed study is needed. Research articles were not found in relation with effects of beet root juice and dysmenorrhoea. Only few studies were conducted among women to know the effect of short-term beet root juice supplementation that too in physical effort. There is minimal research published regarding the effects of intake of beet root juice supplementation among women. None of these studies investigated effects of extract of beet root on menstrual cramps (dysmenorrhea). With that said, there is still a lack of well-conducted human trials Hence further study must be conducted in large number of sample in detailed manner to know the relationship between intake beet root juice and suppression of pain during menstruation.

## Conclusions:

Beetroot is high in beta-carotene and antioxidants, which assist to increase the flow of oxygenated blood to the uterus, which reduces inflammation and relieves the discomfort and severity of menstrual cramps. Data shows that beetroot juice can be utilised as a cost-effective treatment for dysmenorrheal.
